# Highlighting overlooked biodiversity through online platforms: The “Chalcid Wasps of Cyprus” website

**DOI:** 10.3897/BDJ.12.e129367

**Published:** 2024-09-12

**Authors:** Evangelos Koutsoukos, Jakovos Demetriou, Christos Georgiadis, Mircea-Dan Mitroiu, Stephen Compton, Angeliki F Martinou

**Affiliations:** 1 Laboratory of Vector Ecology and Applied Entomology, Joint Services Health Unit Cyprus, BFC RAF Akrotiri BFPO 57, Limassol, Cyprus Laboratory of Vector Ecology and Applied Entomology, Joint Services Health Unit Cyprus, BFC RAF Akrotiri BFPO 57 Limassol Cyprus; 2 Section of Zoology and Marine Biology, Department of Biology, National and Kapodistrian University of Athens, 15784, Athens, Greece Section of Zoology and Marine Biology, Department of Biology, National and Kapodistrian University of Athens, 15784 Athens Greece; 3 Enalia Physis Environmental Research Centre, Acropoleos 2, Aglantzia 2101, Nicosia, Cyprus Enalia Physis Environmental Research Centre, Acropoleos 2, Aglantzia 2101 Nicosia Cyprus; 4 Department of Ecology and Systematics, Faculty of Biology, National and Kapodistrian University of Athens, Athens, Greece Department of Ecology and Systematics, Faculty of Biology, National and Kapodistrian University of Athens Athens Greece; 5 Museum of Zoology, National and Kapodistrian University of Athens, 15784, Athens, Greece Museum of Zoology, National and Kapodistrian University of Athens, 15784 Athens Greece; 6 Alexandru Ioan Cuza University, Faculty of Biology, Iasi, Romania Alexandru Ioan Cuza University, Faculty of Biology Iasi Romania; 7 School of Biology, University of Leeds, Leeds, United Kingdom School of Biology, University of Leeds Leeds United Kingdom

**Keywords:** awareness raising tool, biodiversity, Chalcidoidea, data platform, Hymenoptera, insect conservation

## Abstract

Biodiversity data platforms including databases, websites and data repositories underpin conservation efforts by collecting spatiotemporal data of discovered native and alien species and maps of their distributions. Chalcid wasps (Hymenoptera, Chalcidoidea) are one of the most diverse insect groups estimated to include half a million species. Being mostly parasitoids of other arthropods, they have been successfully used as biological control agents against serious agricultural pests worldwide. In Cyprus, only 124 species of chalcid wasps have been recorded, with 53 species being alien to the island. Their true biodiversity is predicted to be much larger because the island is both under-sampled and under-researched. A number of biodiversity data platforms focusing on the biodiversity of Cyprus are currently online; however, an online platform dedicated on the chalcid wasps of Cyprus is lacking. In the framework of the Darwin Plus Fellowship (DPLUS202) “Species richness and biological invasions of Chalcid wasps in Akrotiri Peninsula”, the “Chalcid wasps of Cyprus” website (https://sites.google.com/view/chalcidwaspscyprus) is presented. This online, dynamic database aims to: (1) raise public awareness regarding a rather neglected and yet ecologically important insect group, (2) provide data on the morphology, ecology and biodiversity of Chalcidoidea on Cyprus, as well as (3) promote conservation needs by setting a baseline for the future assessment of both native and alien chalcid wasp species. This online platform will be regularly revised in order to provide an up-to-date, user-friendly digital environment to the scientific community, policy-makers and citizens.

## Introduction

Biodiversity loss has been increasing alarmingly in the last century and has been identified as one of the top five risks to the global economy, thus underlining the importance of monitoring efforts (Achieng et al. 2023). The growing interest in disseminating biodiversity knowledge to both the scientific community and citizens has led to the creation of a wide variety of biodiversity data platforms (e.g. databases, websites, data repositories and citizen-science platforms) (Borges et al. 2010, Noyes 2019, Kalaentzis et al. 2021, UCD Community 2023). In turn, the accumulation of such data plays a crucial role in mapping species’ distributions and conservation schemes (Jetz et al. 2012, Takashina and Kusumoto 2023). Widely accessible online data have extensively aided efforts in detecting recently introduced species (Ruzzier et al. 2020, Demetriou et al. 2022, Kalaentzis et al. 2023), even generating knowledge-shortfalls regarding the conservation of threatened species (Contreras-Díaz et al. 2023). However, global biodiversity databases, such as GBIF (Global Biodiversity Information Facility), indicate that insects (Insecta) constitute one of the most under-represented classes amongst animals because of their huge biodiversity (Troudet et al. 2017).

Chalcid wasps (Hymenoptera, Chalcidoidea) are one of the most diverse insect groups, recently reclassified into 50 separate families (Burks et al. 2022, Cruaud et al. 2023). With more than 27,000 currently described species worldwide, their true biodiversity is estimated to reach half a million species (Noyes 2000, Heraty 2017, Noyes 2019, van Noort 2024). The majority of chalcid wasps are parasitoids of a wide host-range of host species, including thirteen insect orders and even arachnoids (mites, pseudoscorpions, spiders, ticks) or gall-forming nematodes (Austin et al. 1998, Gibson et al. 1999, Buczek et al. 2021, Cruaud et al. 2023). Nevertheless, representatives belonging to nine chalcid wasp families have evolved secondary phytophagy, including seed-predators, stem-borers, gall-makers, inquilines and entomophytophagous species (Böhmová et al. 2022). Chalcid wasps have been widely used in biological control schemes in order to mitigate the populations of serious agricultural pests, with the most economically important species being distributed within Aphelinidae, Encyrtidae, Eulophidae, Pteromalidae and Trichogrammatidae (Heraty 2017). Overall, hundreds of species of chalcid wasps have been successfully applied in biological control programmes (Heraty 2017, Noyes 2019).

To date, in Cyprus, there has never been a comprehensive study regarding the fauna of chalcid wasps. Noyes (2019) listed a total of only 124 species in 18 families occurring in Cyprus, while Demetriou et al. (2023) reported 53 alien species to the island. The island of Cyprus is situated at the eastern side of the Mediterranean, an area designated by IUCN as a biodiversity hotspot, close to Africa, Asia Minor and the Levantine coast (Valavanidis and Vlachogianni 2013). Great Britain, a northern island with a far smaller insect fauna than that of Mediterranean areas, has more than 1500 chalcid wasp species (Noyes 2019). Turkey and Israel, while larger and continental states, have more than 1000 and 500 species reported, respectively (Noyes 2019). Therefore, given these numbers, as well as ongoing literature review and collecting efforts, indicate that the present number of species greatly underestimate the true chalcid wasp biodiversity in Cyprus, even though recent studies have added both new native and alien species to the checklist of the island (Japoshvili et al. 2023, Koutsoukos et al. 2024). While there is a variety of biodiversity data platforms focusing on the biodiversity of Cyprus, such as BirdLife Cyprus (https://birdlifecyprus.org/), the Flora of Cyprus (Hand et al. 2011), the Cyprus Database of Alien Species (CyDAS) (Martinou et al. 2020), The Cyprus Atlas of Reptiles and Amphibians (the Cyprus Herp Atlas) (Zotos et al. 2023), the Alien to Cyprus Entomofauna (ACE) database (Demetriou et al. 2023) and Biodiversity of Cyprus (https://biodiversitycyprus.blogspot.com/), a portal providing information and data on the chalcid wasps of Cyprus as a whole is lacking. Nevertheless, the monumental work of Noyes (2019) (Universal Chalcidoidea Database) and its new version (https://ucd.chalcid.org/) constitute an important baseline in any efforts for studying chalcid wasps on the island.

The “Chalcid wasps of Cyprus” website aims to pool and present all available knowledge regarding the biodiversity of native and non-native chalcid wasps found in Cyprus, their distribution, ecology, host associations, taxonomy and historical data on the Chalcidoidea fauna of the island. This online platform will be regularly updated in order to provide an up-to-date, user-friendly digital environment to both the scientific community and citizens. The website’s main objectives are:

1. To raise public awareness regarding a rather neglected and yet ecologically important insect group.
2. Provide information on the morphology (photographic material), ecology (distribution, host-associations), biodiversity (dynamic species checklists of native and alien species) and available scientific knowledge on the chalcid wasps present on Cyprus.
3. Highlight conservation needs by setting a baseline for the future assessment of both native (based on IUCN categories and criteria) and non-native or alien chalcid wasp species [against the Environmental Impact Classification for Alien Taxa (EICAT) and Socio‐Economic Impact Classification of Alien Taxa (SEICAT) protocols] (Blackburn et al. 2014, Bacher et al. 2018), based on distributional data and host-associations.

## Materials and Methods

### Construction of website

The “Chalcid wasps of Cyprus” website (https://sites.google.com/view/chalcidwaspscyprus) was developed on “Google Sites” under the project DPLUS202 “Species richness and biological invasions of Chalcid wasps in Akrotiri Peninsula” (https://www.darwininitiative.org.uk/project/DPLUS202/), funded by the Darwin Initiative – Biodiversity Challenge Funds. The Gmail account (chalcidwaspscy@gmail.com) was set up for the website, with its provided Google Drive file storage hosting all available photographic material, documents, sheets and data. The platform has been made available online upon the submission of the manuscript and paired with Google Analytics to monitor website traffic.

### Construction of species profiles

Species taxonomy follows Burks et al. (2022). The status of each species is assessed as either “native”, “native (endemic)”, “alien (intentionally introduced)” or “alien (unintentionally introduced)”. Species stated as “alien” represent non-native species introduced via human activities outside their native range (EU 1143/2014). In the case of alien species, their origin in terms of biogeographic realms (Afrotropical, Australasia, Indomalaysian, Nearctic, Neotropical, Palearctic) is provided. The alien, cryptogenic or questionable status of species was determined through available species checklists and databases on a European level, as well as experts’ communication and available scientific literature (DAISIE 2009, Rasplus et al. 2010, EASIN 2021, Demetriou et al. 2023).

Regarding the distribution of species, for each one, their worldwide distribution in terms of biogeographic realms is also provided. On a local scale (where available), distributional maps are provided for each species, depicting its distribution within district level (Akrotiri UK SBA, Famagusta, Dhekelia UK SBA, Kyrenia, Larnaca, Limassol, Nicosia, Paphos). In cases where data are insufficient (e.g. “Cyprus” in general or no data are provided), maps were not generated.

The ecology of species was assessed as either “parasitoid” or “phytophagous” presenting data on associated plant- or animal-hosts in a global and local scale, following UCD Community (2023).

Regarding conservation, threats classification scheme follows IUCN (https://www.iucnredlist.org/resources/threat-classification-scheme) and presence in protected areas refers to the designated NATURA 2000 network sites on the island (https://natura2000.eea.europa.eu/) (92/43/EEC; 2009/147/EC), as well as the Akrotiri Peninsula RAMSAR site (site number: 1375) (https://rsis.ramsar.org/ris/1375).

On the bottom of each species profile page, relevant scientific literature is provided.

### Website overview

**Figure 1.**
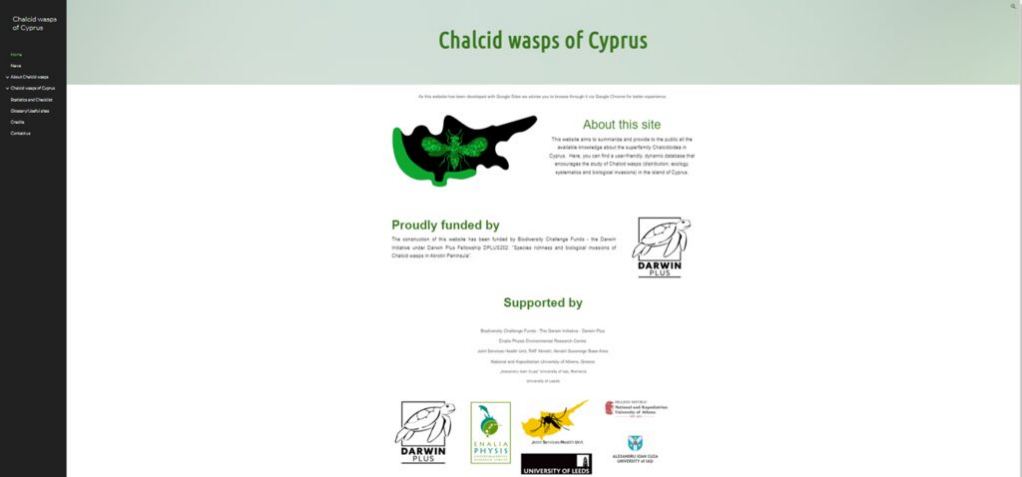
The front page of the Chalcid wasps of Cyprus website.

The main menu of the site is divided into the following top-level pages:

**Home.** Welcome page stating the funding declaration and the website’s main objectives (Fig. 1).

**News.** Page presenting new publications concerning the Chalcidoidea of Cyprus, events, citizen-science schemes, conferences and workshops attended, press releases etc.

**About Chalcid wasps.** A menu section page further divided into the following pages: “Classification”, “Ecology” and “Biological invasions” providing introductory information to the wider public. In the latter page, an up-to-date checklist of alien Chalcidoidea of Cyprus is also provided.

**Chalcid wasps of Cyprus.** A menu section in the form of a taxonomic backbone with information on the families > subfamilies > genera > and species present on the island (number of representatives in subfamilies, genera and species given in parentheses). On a species level, profiles for all species found in Cyprus are provided including data on their status, distribution, year of first record, ecology, known hosts/associates (globally and locally), conservation and relevant scientific literature accompanied by photographic material and distributional maps.

**Statistics and checklist.** A page providing up-to-date statistics on the number of families, genera and species present on Cyprus, including the number of endemic and alien species and their respective percentages over the total chalcid wasp fauna of the island. An up-to-date checklist of species is provided denoting endemic and alien taxa.

**Glossary/Useful sites.** Providing index to used abbreviations and useful sites/sources (i.e. Chalcid.org contributors 2018, Noyes 2019, UCD Community 2023, van Noort 2024, van Noort and Rasplus 2024).

**Credits.** Listing the project members, acknowledging and listing data contributors and supporting organisations.

**Contact us.** Includes the website’s citation and contact information (chalcidwaspscy@gmail.com).

## Data usage

### Awareness-raising

Despite their immense diversity and ecological significance, chalcid wasps are both understudied and overlooked as indicated by Weber et al. (2018), pointing out problems due to their minute size, the lack of taxonomists and taxonomic revisions on genera and species, shortfalls in literature on known host-associations, as well as the relevant ease with which joint efforts of citizen and scientists can unravel new national records of species. Throughout the created species profiles, checklists and introductory information on the taxonomy, ecology and biological invasions of chalcid wasps, the “Chalcid wasps of Cyprus” website aims to accumulate and present all available scientific data of the chalcid wasps that are present on Cyprus, supplementing ecological shortfalls (Hortal et al. 2015) showcasing the species richness of a biodiversity hotspot area (Myers et al. 2000) and bringing information to the public and policy-makers regarding this neglected insect group of parasitoid wasps (Weber et al. 2018). Such data can be of use not only to taxonomic specialists working on parasitoid wasps, but also to ecologists studying species interactions, conservation practitioners, policy-makers, education professionals and nature enthusiasts or citizen scientists.

### Taxonomy

With an estimated species richness of around half a million species and the constant rise of taxonomic revisions, the biodiversity and taxonomy of chalcid wasps should be rigorous and particularly older records should be verified to provide reliable and complete species checklists (Weber et al. 2018). The dynamic “Chalcid wasps of Cyprus” website aims to provide an up-to-date checklist including statistics on the number of genera, species, endemic and alien taxa (see Statistics and Checklist page). On the one hand, based on the data available, only three endemic species are known from Cyprus, with this number perceived as extremely low and contradicting the characterisation of Cyprus as a “biodiversity hotspot”. On the other hand, the number of alien species (53), compared to the total known chalcid wasp species of Cyprus (124) is excessive. A percentage of almost 43% alien species clearly shows that the Chalcidoidea fauna of Cyprus is largely unknown. Data on new records and species, developed through Darwin Fellowship (DPLUS202), will be made available online as soon as these are published, supplementing the provided metrics and species profiles.

### Ecology and conservation

On each species profile, the status, distribution, year of first record, ecology and known hosts/associates are provided. Such can be utilised towards constructing the invasion history and spread of alien species intentionally released or accidentally introduced and subsequently detected, as well as assessing their impacts on native biodiversity and integration in Mediterranean ecosystems, especially within protected areas. As for native and endemic taxa, knowledge on species interactions can help us better predict and map the distribution of both host plants, animals and their parasitoids, shed light on unknown links in ecological networks, as well as assessing any cascading effects of biodiversity loss on other trophic levels. Acknowledging the presence of species in protected areas is important for both conservation practitioners and for preserving taxonomic diversity in an era of increasing urbanisation and habitat loss, while the provided data can also serve as a backbone for the future assessment of species under categories and criteria of IUCN as no species of chalcid wasps have been evaluated.
